# Seasonal and daily variations in primary and secondary metabolism of three maquis shrubs unveil different adaptive responses to Mediterranean climate

**DOI:** 10.1093/conphys/coz070

**Published:** 2019-11-05

**Authors:** Antonella Gori, Massimiliano Tattini, Mauro Centritto, Francesco Ferrini, Giovanni Marino, Jacopo Mori, Lucia Guidi, Cecilia Brunetti

**Affiliations:** 1 Department of Agriculture, Food, Environment and Forestry, University of Florence, viale delle Idee 30, 50019, Sesto Fiorentino, Florence, Italy; 2 Institute for Sustainable Plant Protection, National Research Council of Italy, via Madonna del Piano 10, 50019, Sesto Fiorentino, Florence, Italy; 3 Department of Agriculture, Food and Environment, University of Pisa, Lungarno Pacinotti 43, 56126, Pisa, Italy

**Keywords:** Abscisic acid, coastal dune ecosystems, gas exchange, maquis species, Mediterranean climate, photosynthetic pigments, polyphenols, water relations

## Abstract

Maquis species play a central role in the maintenance of coastal ecosystems thanks to anatomical, physiological and biochemical features evolved to cope with severe stress conditions. Because the seasonal and daily dynamics of physiological and biochemical traits of maquis species are not fully addressed, we performed a field study on three coexisting Mediterranean shrubs (*Pistacia lentiscus* L. and *Phillyrea latifolia* L., evergreen schlerophylls, and *Cistus incanus* L., semi-deciduous) aiming at detecting the main adaptive differences, on a seasonal and daily basis, in primary and secondary metabolism along with the principal climatic determinants. These species differed in their physiological and biochemical responses especially on a seasonal level. In *P. latifolia*, a great investment in antioxidant phenylpropanoids contributed to maintain high photosynthetic rates throughout the whole growing season. In *C. incanus*, high carotenoid content associated with chlorophyll (Chl) regulation alleviated oxidative damage during the hot and dry summers and help recover photosynthesis in autumn. In *P. lentiscus*, high abscisic acid levels allowed a strict control of stomata, while fine Chl*a*/Chl*b* regulation concurred to avoid photoinhibition in summer. Temperature resulted the most important climatic factor controlling the physiological and biochemical status of these coexisting shrubs and, thus, in determining plant performances in this Mediterranean coastal habitat.

## Introduction

The Mediterranean basin is a recognised biodiversity hotspot, where 10% of the world’s higher plants can be found in an area representing only ~ 2% of the Earth’s surface (Médail and Quézel, 1997; Myers *et al*., 2000), as well as a climate change hotspot, affected not only by above-global average temperature increase and precipitation reduction but also by increasing occurrence of heat waves associated with severe droughts (Barriopedro *et al*., 2011; Hewitson *et al*., 2014; Samaniego *et al*., 2018). These climatic changes along with other anthropogenic ‘forcing’, such as urbanization, grazing and intensive agriculture practices, are resulting in fragmentation of the Mediterranean maquis, especially in coastal areas (Bellard *et al*., 2014; Matesanz and Valladares, 2014), and, in turn, in degradation of the ecosystem services that maquis vegetation produces (Centritto *et al*., 2011; Maestre *et al*., 2012, Maestre *et al*., 2016).

Maquis evergreen sclerophyll bushes and small size semi-deciduous shrubs play a vital role in the maintenance and preservation of coastal dune ecosystems (Valencia *et al*., 2015; Drius *et al*., 2016), possessing a series of constitutive traits to cope successfully with severe environmental stresses (Domínguez *et al*., 2012). This Mediterranean coastal vegetation has been included in different functional classifications based on the species morphological traits and water-use behaviours (Galmés *et al*., 2007; Hernández *et al*., 2010). Evergreen sclerophylls face drought conditions with a high specific leaf area, thick cuticle and deep root system (Karavatas and Manetas, 1999). Whereas, semi-deciduous species partially avoid water stress through a reduction of their foliage area, thus restricting their growth to the more favourable seasons (Werner *et al*., 1999; Oliveira and Peñuelas, 2005). In addition, Mediterranean plants can be also classified as drought avoiding and drought-tolerant species based on their physiology (Lo Gullo and Salleo, 1988). In this sense, drought avoiding plants undergo limited changes in leaf water potential and/or relative water content (RWC) during water stress. This is achieved by either restricting water loss from the plant body (water saving) or by increasing water absorption to replace losses by transpiration (water spending) (Kozlowski and Pallardy, 2002). By contrast, drought-tolerant plants can survive at low water potentials maintaining high RWC (drought-tolerance dehydration-avoidance) or tolerate low RWC (drought-tolerance dehydration-tolerance) (Kozlowski and Pallardy, 2002). This classification roughly corresponds to the isohydric/anisohydric terminology (*sensu*Tardieu and Simonneau, 1998), in which isohydric plants are described as capable of maintaining constant daily minimal leaf water potential (*Ψ*_w_) regardless of soil water potential, while the anisohydric plants show progressively lower *Ψ*_w_ as a function of decreasing soil water availability (Nardini *et al*., 2014; Hochberg *et al*., 2017). However, these behaviours are not mutually exclusive and, in practice, plants may switch from isohydric to anisohydric, depending on the severity of drought (Domec and Johnson, 2012).

Mediterranean maquis plants also have the capacity of fine-tuning the biosynthesis of a huge variety of secondary metabolites, which can underlie an impressive multiplicity of protective roles because of their large diversity of chemical structures. Among these adjustments, the increase in carotenoid and polyphenol contents in stressed plants has been linked to improvements in photoinhibition tolerance and, in general, in the protection of photosynthetic organs from photo-oxidative damage (Peñuelas and Munné-Bosch, 2005; Hernández *et al*., 2012; Selmar and Kleinwächter, 2013; Brunetti *et al*., 2015). In particular, the xanthophyll cycle pigments protect photosystem II (PSII) by dissipating as heat the excess of light energy (non-photochemical quenching [NPQ]) (Demmig-Adams and Adams, 1996). Moreover, adjustments in photosynthetic pigment composition, such as decreasing the total chlorophyll content or increasing the ratio of violaxanthin-cycle pigments to total chlorophyll (Logan *et al*., 1998; Havaux and Tardy, 1999; Lu *et al*., 2003), may reduce the risk of photodamage and limit lipid peroxidation (Esteban *et al*., 2015; García-Plazaola *et al*., 2017). Similarly, polyphenols, and in particular phenylpropanoids, display a general protective and antioxidant function, depending on their chemical features and their location in the leaf (Agati *et al*., 2012). For example, UV-absorbing flavonoids in the epidermal cells strongly attenuate highly energetic solar wavelengths, thus reducing photo-oxidative stress (Hernández *et al*., 2009). Moreover, mesophyll-located flavonoids may complement the function of primary antioxidants maintaining whole-cell ROS levels within a sub-lethal concentration range (Agati and Tattini, 2010).

Besides the aforementioned antioxidants, plants adjust leaf abscisic acid (ABA) levels depending on stress conditions (Nambara and Marion-Poll, 2005). This plant hormone is known for its function in the regulation of stomatal closure in the guard cells resulting in declines in transpiration and consumption of water (Zhu, 2002; Zhang *et al*., 2006). In addition, ABA is involved in the activation of the antioxidant metabolism, triggering stress-related gene expression, thus conferring tolerance to drought (Lu *et al*., 2009; Lim *et al*., 2015; Li *et al*., 2017).

Mediterranean coastal dunes represent critical and vulnerable habitats, characterised by the coexistence of different plant communities in a relatively small area (Acosta *et al*., 2009; Fenu *et al*., 2013). In order to protect and preserve this ecosystem, it is essential to compare the main response strategies of native plants and to select appropriate meaningful traits linked to specific climatic factors, especially in the context of ongoing climate change. In addition, field studies on seasonal and daily dynamics of physiological and biochemical traits of coexisting maquis species are still lacking (Fernández-Marín *et al*., 2017).

Here we present a comparative study, performed under natural conditions, on three widespread and co-occurring species of Mediterranean maquis: two evergreen schlerophylls, *Pistacia lentiscus* L. and *Phillyrea latifolia* L., and the semi-deciduous *Cistus incanus* L. (sin. *Cistus x incanus* L.). These species have been previously classified on the basis of their different water-use behaviours and gas exchange performances: *P. latifolia* is a drought-tolerant species (Barbeta *et al*., 2015), *P. lentiscus* is a drought avoider-water spender (Ozturk *et al*., 2010; Trifilò *et al*., 2015), and *C. incanus* is a drought avoider-water saver plant (Werner *et al*., 1999; Sánchez-Blanco *et al*., 2002). Physiological and biochemical traits were monitored *in situ* on a daily and seasonal basis during two consecutive years, and their relationships with key climatic factors (i.e. precipitation, irradance and air temperature) were evaluated. This study aimed at investigating the main differences in primary and secondary metabolism and identifying the principal climatic factors affecting these functional traits in their natural habitat.

## Materials and methods

### Plant material, study area and experimental design

The study was performed in 2014 and 2015 on the coastal dunes of Southern Tuscany, Italy. The experiment was located in a coastal sand-dune area of about 200 m^2^ at 42° 46′N, 10°53′E (mean annual temperature, 15.2 °C; annual precipitation, 620 mm), where there were more than 30 individual plants of *Pistacia lentiscus* L., *Phillyrea latifolia* L. and *Cistus incanus* L. Four homogeneous plants per species were chosen randomly in the selected area, tagged and used as replicates for all physiological and biochemical measurements throughout the growing season. The sampling was performed at the branch scale at the top of the canopy. *P. latifolia* and *P. lentiscus* individuals were about 1.2 to 1.5 m height with a canopy area of 1 to 1.2 m^2^, whereas *C. incanus* plants were about 0.6 m high with a canopy area of 0.6 to 0.7 m^2^. Diurnal courses of leaf water potential and osmotic potential (five different sampling hours from pre-dawn, PD: 4:00 a.m., 8:00 a.m., 12 noon—midday, MD—3:00 p.m. and 6:00 p.m.), as well as gas exchange, chlorophyll fluorescence parameters and metabolite analyses (four times during the day: 8:00 a.m., 12 noon, 3:00 p.m. and 6:00 p.m.) were performed on cloudless days in spring (27–29 May 2014 and 29–30 May 2015), summer (03–06 July 2014 and 07–09 July 2015) and autumn (04–06 October 2014 and 2015). Air temperature (T), precipitation (P) and global irradiance (GI) (measured in the 200–3000 nm range of solar wavebands) during the whole experimental period were recorded every hour by the weather station ‘Ponti di Badia’, located 7 km from the study site.

### Physiological measurements (water relations, gas exchange and chlorophyll fluorescence)

Leaf water potential (*Ψ*_w_) and osmotic potential (*Ψ*_π_) were measured on two leaves per plant using a Scholander-type pressure chamber (PMS Instruments, Corvallis, OR) and a boiling point Wescor VAPRO 5520 osmometer (Wescor Inc., Logan, UT), respectively. The water relations values measured on leaves of the same plant were combined to make an individual replicate.

The difference between midday (*Ψ*_wMD_) and pre-dawn water potential (*Ψ*_wPD_) was calculated as Δ*Ψ_w_* = *Ψ*_*π*MD_ − *Ψ*_*π*PD_. Similarly, the difference between midday and pre-dawn water potential (*Ψ*_wPD_) was calculated as Δ*Ψ_π_* = *Ψ*_*π*MD_ − *Ψ*_*π*PD._

Net photosynthesis (*P*_n_) and stomatal conductance (*g*_s_) were measured on fully expanded, sunny-exposed leaves of the upper part of the crown using a LI-6400 portable photosynthesis system (Li-Cor, Lincoln, NE, USA), with a cuvette size of 2 cm^2^ and operating at ambient [CO_2_] and at the photosynthetic photon flux density (PPFD) recorded in the environment. Intrinsic water use efficiency (WUEi) was calculated as the ratio between *P*_n_ and *g*_s_.

Chlorophyll fluorescence was measured using a portable PAM-2000 Chl fluorometer (Heinz Walz, Effeltrich, Germany). Maximum photochemical efficiency of photosystem II (F_v_/F_m_) was measured in 20 min dark-adapted leaves as F_v_/F_m_ = (F_m_ − F_0_)/F_m_, where F_v_ (variable fluorescence) is calculated as the difference between F_m_ (maximal fluorescence—measured in ~ 0.8 s with a saturating PPFD pulse of 8000 μmol m^−2^^−1^) and F_0_ (minimum fluorescence—measured using low PPFD of ~ 1 μmol m^−2^ s^−1^). Leaves were then exposed to actinic light and a second saturating pulse was applied to determine the maximum fluorescence in light-adapted state (F_m_′) and the steady-state fluorescence (F_s_). Then NPQ was calculated as NPQ = (F_m_ − F_m_′)/F_m_′ (Schreiber *et al*., 1986), whereas actual efficiency of PSII was calculated as Φ_PSII_ = (F_m_′ − F_s_)/F_m_*′* (Genty *et al*.*,* 1989).

### Biochemical analyses

Leaf samples were collected, immediately frozen in liquid nitrogen, stored at −80 °C and then lyophilized. Then, secondary metabolites and hormones were quantified on a dry weight (DW) basis. To measure photosynthetic pigments and polyphenols, lyophilized material (150 mg) was extracted with 2 × 2.5 mL acetone (with the addition of 0.5 g L^−1^ CaCO_3_) and injected (15 μL) into a Perkin Elmer Flexar liquid chromatograph equipped with a quaternary 200Q/410 pump and an LC 200 diode array detector (DAD) (all from Perkin Elmer, Bradford, CT). Photosynthetic pigments were separated in an Agilent Zorbax SB-18 (250 × 4.6 mm, 5 μm) thermostated at 30 °C using an 18-minute run and a linear gradient solvent system from 100% of solvent A (methanol/water, 95/5) to 100% solvent B (methanol/ethylacetate, 6.8/3.2) with a flow rate of 0.8 mL min^−1^. Individual carotenoids and chlorophylls were identified and quantified using retention times and UV spectral characteristics of authentic standards from extrasynthese (Lyon-Nord, Genay, France) and calculated on DW basis using RWC data. VAZ and de-epoxidation state of the xanthophyll cycle (DES) were calculated as: VAZ = V + A + Z and DES = (A + Z)/(A + Z + V), where V*,* A and Z represent violaxanthin, antheraxanthin and zeaxanthin concentrations, respectively. Individual polyphenols were identified and quantified using HPLC-DAD analysis. In detail, lyophilized material (150 mg) was extracted twice with 5 mL of ethanol/water (75/25) adjusted at pH 2.5 with formic acid and the supernatant partitioned with 3 × 5 mL of *n*-hexane. The ethanol fraction was reduced to dryness, and the residue was rinsed with 1 mL of methanol/water (90/10). Aliquots of 10 μL were injected into the Perkin Elmer liquid chromatography unit reported earlier. Phenylpropanoids were separated using a Agilent Zorbax SB-18 (250 × 4.6 mm, 5 μm), operating at 30 °C with a flow rate of 1 mL min^−1^ and eluted with a linear gradient solvent system from 100% solvent A (water adjusted to pH 2.5 with HCOOH/acetonitrile [90/10]) to 100% solvent B (acetonitrile/water adjusted to pH 2.5 with HCOOH [90/10]) over a 45-minute run. Identification and quantification of these metabolites was carried out using retention times and UV spectral characteristics of authentic standards, as well as based on literature data and reported on DW basis as total phenylpropanois (PP_Tot_) and total polyphenols (POL_Tot_). In particular, in *C. incanus* leaves, PP_Tot_ were constituted by flavonol glycosydes (i.e. myricetin and quercetin glycosides), whereas POL_Tot_ were represented by the sum of condensed tannins (proanthocyanidins) and flavonol glycosides. In *P. lentiscus*, PP_Tot_ were constituted by flavonol glycosydes (mainly myricetin derivatives) and gallic acid derivatives, while POL_Tot_ were composed of hydrolisable tannins (galloyl derivatives of quinic acid) and flavonol glycosides. No detectable levels of tannins were found in leaves of *P. latifolia*. Thus, in this species, the sum of total polyphenols (POL_Tot_) corresponds to the total concentration of phenylpropanoids (PP_Tot_), which are composed of flavonol glycosides (i.e. quercetin and luteolin glycosides) and hydroxycinnamic acid derivatives (mostly caffeic acid derivatives). Analyses of ABA and ABA glucose ester (ABA-GE) were performed on lyophilized leaf material (150 mg) ground in liquid nitrogen and added with 40 ng of deuterium-labeled internal standards (*d*_6_-ABA and *d*_5_-ABA-GE from the National Research Council of Canada). Then ABA and ABA-GE were extracted with 3 × 1 mL pH 2.5 CH_3_OH/H_2_O (50/50), at 4 °C for 30 minutes. The supernatant was defatted by N-hexane extraction (2 × 3 mL) and purified through Sep-Pak C18 cartridges (Waters, MA), eluted with 1.2 mL of ethylacetate. Then, the eluate was reduced to dryness under nitrogen and rinsed with 250 μL of CH_3_OH/H_2_O (50/50). Finally, 3 μL of sample solution were injected into the LC–ESI–MS/MS system consisting of a UPLC (Nexera UPLC Shimadzu Corporation) coupled with a MS/MS detector (TQ 8030) equipped with an ESI source (all from Shimadzu Corporation, Kyoto, Japan) operating in negative ion mode. Compounds were separated using a Poroshell C18 column (3.0 × 100 mm, 2.7 μm i.d.; Agilent, USA). Gradient elution was performed with water acidified with 0.1% formic acid (solvent A) and acetonitrile/methanol (1/1) with the addition of 0.1% of formic acid (solvent B) at a constant flow-rate of 300 μL∙min^−1^ ranging from 95% solvent A to 100% solvent B during a 30-minute run. Quantification was conducted in multiple reaction mode (MRM, López-Carbonell *et al*., 2009).

### Statistical analysis

Data were subjected to a two-way repeated-measures analysis of variance (RP-ANOVA), where ‘species’ and ‘season’ were the between subject factor and ‘sampling hour’ was the within-subject factor (each hour as a single level for a total of 4 levels) (SPSS v.20; IBM, Chicago, IL, USA). Mean values were separated by using Tukey’s *post hoc* test (*P* ≤ 0.05) after having checked the normality and homoscedasticity of the dataset. Since significant interactions between ‘species’ and ‘season’ as well as between ‘species’ and ‘sampling hour’ occurred, we performed a one-way ANOVA followed by Tukey’s *post hoc* test (*P* ≤ 0.05) to evaluate the effect of ‘season’ and ‘sampling hours’ on species separately. In addition, we estimated the effect of the temporal factors and their interaction with species on biochemical and physiological traits, throughout the eta-squared value (*η*^2^):

$$ {\eta}^2={\mathrm{SS}}_{\mathrm{factor}}/\left({\mathrm{SS}}_{\mathrm{factor}}+{\mathrm{SS}}_{\mathrm{residual}}\right) $$

where *η*^2^ indicates how much of the observed variation (i.e. SS_total_, total sum of squares = SS_factor_ + SS_residual_) can be explained statistically by a factor or an interaction under consideration (SS_factor_; i.e. ‘season’ and ‘sampling hour’, and their interaction with species) (Nakagawa and Cuthill, 2007).

Multiple regression analyses (MRA) were performed for each species to investigate the influence of climatic variables (air temperature, global irradiance and precipitation) on the measured parameters. Linear regression analysis was used to assess possible relationships between different physiological and biochemical traits. Regression coefficients (*r*^2^) were obtained from this analysis to indicate the magnitude of these relationships. Pearson product moment correlation coefficients (R) were used to calculate the degree of correlation among the examined parameters. Principal Component Analyses (PCA) were made on physiological and biochemical data for each season. PCA and MRA were made using STATGRAPHICS Centurion XV.II (StatPoint Inc., Warrenton, Virginia, USA). Graphics were designed using SigmaPlot 12.5 (Systat Software Inc., San Jose, California, USA).

**Figure 1:**
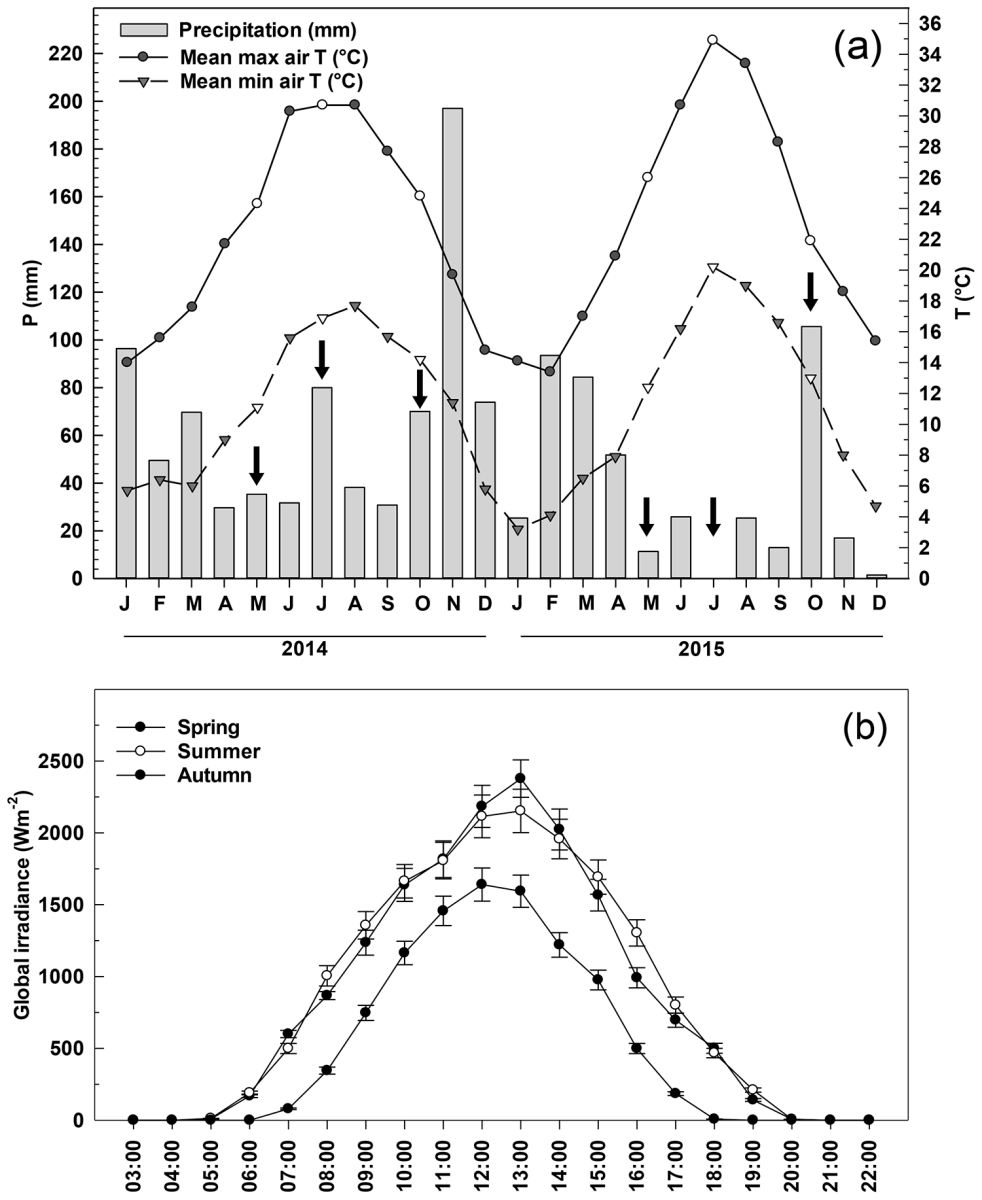
Monthly total precipitation (mm), daily average of maximum and minimum air temperature in 2014 and 2015 (a) (arrows indicate the sampling months); year averages of daily global irradiance (W m^−2^) during the days of measurements (b) (data of the Meteorological Station of Ponti di Badia, Grosseto).

## Results

### Meteorological data

The years 2014 and 2015 were characterized by contrasting rainfalls, as 2015 resulted considerably drier compared to the previous year especially during the summer season (Fig. 1a). In 2014, the cumulative rainfall during the 2 months preceding the measurements was 65 mm between April and May, 67 mm from June to the beginning of July, and 70 mm between August and September. In 2015, the rainfall values in the corresponding periods were 63 mm, 37 mm and 38 mm, respectively. In general, the 2015 growing season also showed higher minimum and maximum temperatures compared to 2014. During the measurement days, the minimum and maximum temperatures were 11.1 °C and 24.3 °C in May, 17.9 °C and 30.7 °C in July and 15.7 °C and 27.7 °C in October in 2014; while in 2015 the minimum and maximum temperatures were 12.4 °C and 26 °C in May, 20.2 °C and 32.9 °C in July, 16.6 °C and 27.3 °C in October 2015. There were no differences in the daily global irradiance (mean of 2014 and 2015) during the days of measurements between May and July, whereas in October global irradiance declined significantly (Fig. 1b).

### Physiological and biochemical traits

All physiological and biochemical parameters were significantly different both on daily and seasonal timescales (*p* < 0.05), with the exception of Chl*a*/Chl*b,* which resulted in no significant effect in ‘sampling hour’ (Table 1). Similarly, all interactions ‘species–season’ and ‘species–sampling hour’ were highly significant (*p* < 0.05), except for ‘species–sampling hour’ of VAZ/Chl_Tot_ which was not significant (Table 1). For most of the physiological (*P*_n_, *g*_s_, *Ψ*_w_, *Ψ*_π_) and biochemical traits (Car_Tot_, Chl_Tot_, VAZ/Chl_Tot_, Chl*a*/Chl*b*, PP_Tot_, POL_Tot_, ABA and ABA-GE), the interaction ‘species–season’ had higher values of eta-squared (*η*^2^) compared to ‘species–sampling hour’, suggesting that, for these parameters, species were mostly differentiated on a seasonal basis (Tab. 1). In contrast, for chlorophyll fluorescence (F_v_/F_m,_ Φ_PSII,_ NPQ) and DES, the differentiation among species was mostly driven by the hour of sampling (highest values of *η*^2^ for ‘species–sampling hour’ interaction) (Tab. 1).

There were clear differences in the gradient of water (*ΔΨ*_w_) and osmotic potential (*ΔΨ*_π_) between MD and PD among the three maquis species (Table 3). *P. latifolia* showed the highest *ΔΨ*_w_ and *ΔΨ*_π_ in all seasons, while *P. lentiscus* the lowest. In addition, in *P. latifolia*, the *ΔΨ*_w_ and *ΔΨ*_π_ increased significantly from spring to summer and decreased in autumn, whereas in the other two species, both *ΔΨ*_w_ and Δ*Ψ*_π_ did not change throughout the seasons (Tab. 3).

**Figure 2:**
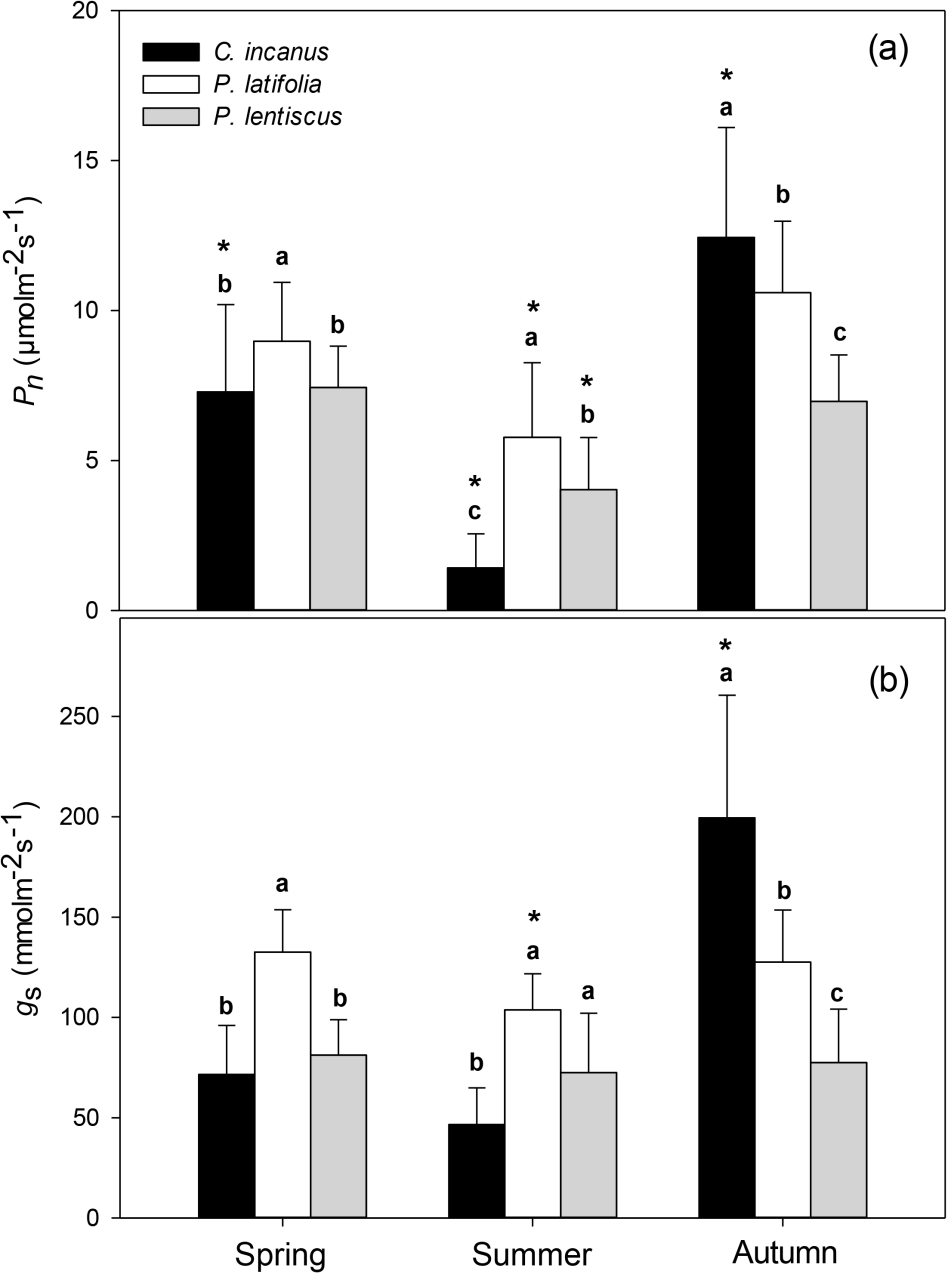
Seasonal trends of (a) net photosynthetic rate (*P*_n_) and (b) stomatal conductance (*g*_s_) in *C. incanus*, *P. latifolia* and *P. lentiscus*. Data are means ± SD (n = 32). Letters indicate significant differences (*p ≤* 0.05) among species for each season, whereas asterisks indicate significant differences (*p ≤* 0.05) among seasons for each species.

**Figure 3:**
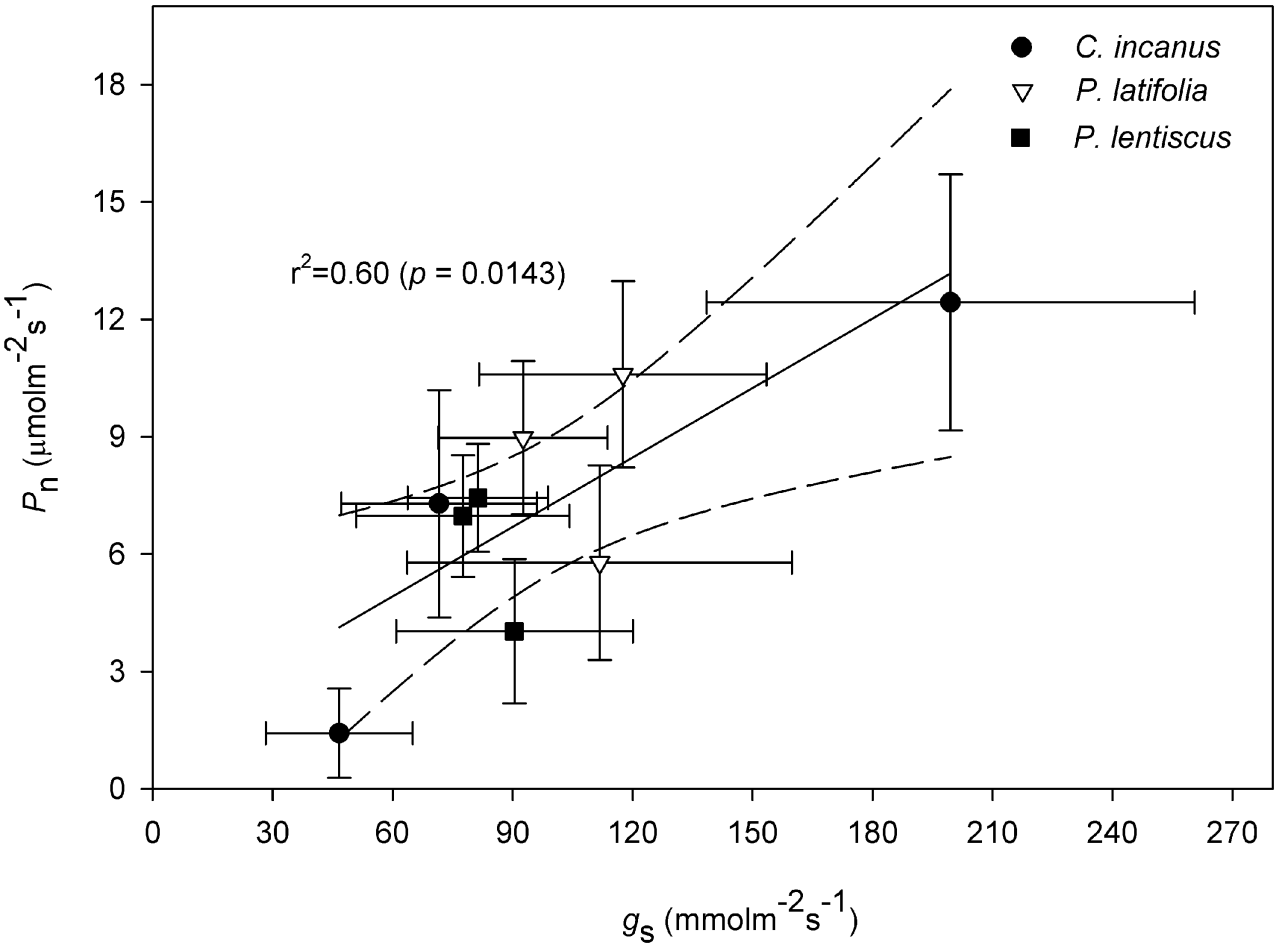
Relationship between net photosynthetic rate (*P*_n_) and stomatal conductance (*g*_s_) in *C. incanus, P. latifolia* and *P. lentiscus* measured in spring, summer and autumn. Dara are means ± SD (n = 9). The central black line indicates the line of best-fit. The dotted lines either side of the best-fit line indicate 95% confidence intervals of the mean. *P* and *r*^2^ values indicate the results of linear regression.

**Figure 4:**
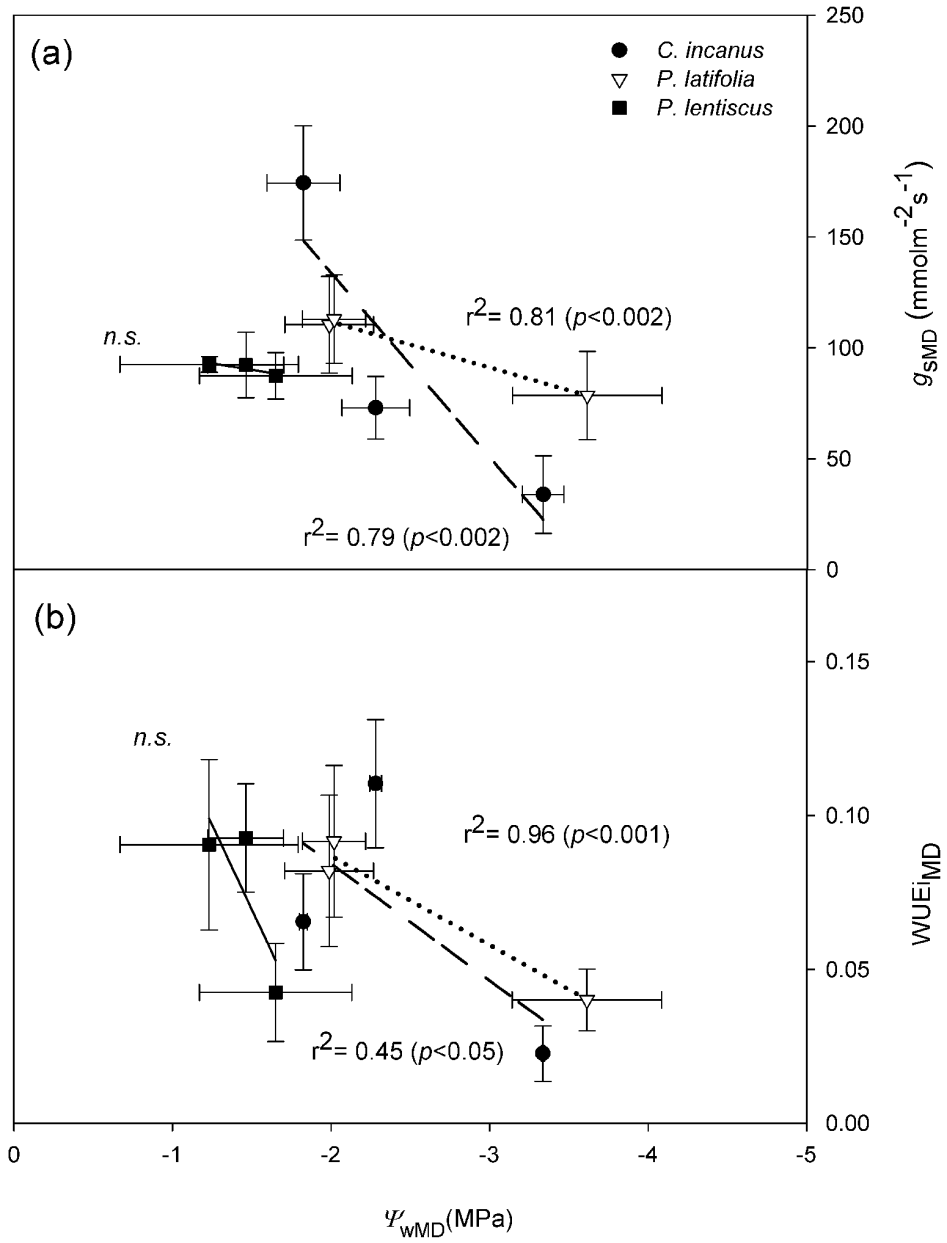
Relationship between midday water potential (*Ψ*_wMD_) and midday stomatal conductance (*g*_sMD_) (a), and between midday water potential (*Ψ*_wMD_) and midday intrinsic water use efficiency (WUEi_MD_) (b) in *C. incanus*, *P. latifolia* and *P. lentiscus* in spring, summer and autumn. Dara are means ± SD (n = 3). The lines indicate the best-fit for the three species; *p* and *r*^2^ values indicate the results of linear regression.

All the species displayed higher *P*_n_ in the spring compared to summer, with *P. latifolia* showing significantly higher values than *C. incanus* and *P. lentiscus.* The summer reductions in *P*_n_ were species-specific (Fig. 2a). Particularly, *P. latifolia* showed the significantly lowest (−35%), whereas *C. incanus* showed the significantly highest (−80%) reduction in *P*_n_, respectively. Towards the end of the growing season, *P*_n_ recovered in all three species. However, the recovery in *P*_n_ was particularly stimulated in *C. incanus*, which showed significantly higher values than *P. latifolia* and *P. lentiscus* was also compared with its *P*_n_ value observed in spring. Photosynthesis also resulted significantly higher in *P. latifolia* than in *P. lentiscus*; however, the *P*_n_ values in these two species were not statistically different from those shown in spring (Fig. 2a). In general, the seasonal course of *g*_s_ had a very similar pattern to that of *P*_n_ in the three species (Fig. 2b, Fig. 3). By contrast, the relationships between *g*_s_ and *Ψ*_w_ at midday showed that the response curves were different among species and a not significant linear relationship was found for *P. lentiscus* (Fig. 4a). When *Ψ*_wMD_ was plotted against WUEi calculated at midday (WUEi_MD_), differences were observed among the shrubs, and this relationship showed the highest *r*^2^ for *P. latifolia* and *C. incanus* (Fig. 4b).

**Figure 5:**
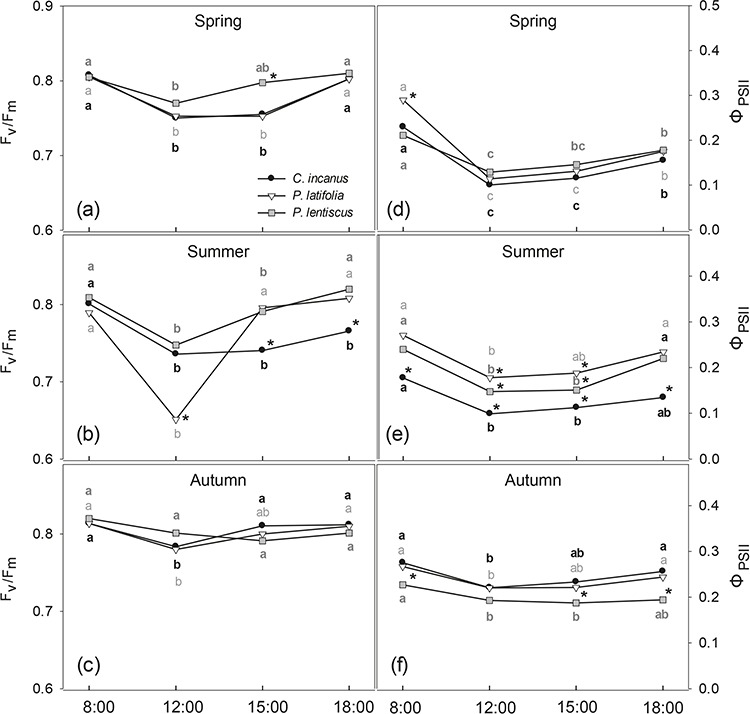
Diurnal trends of maximal efficiency of maximum photochemical efficiency of PSII (F_v_/F_m_) and actual efficiency of the PSII (Φ_PSII_) measured in spring (a, d), summer (b, e) and autumn (c, f) in *C. incanus*, *P. latifolia* and *P. lentiscus*. Data are means ± SD (n = 8). Letters indicate significant differences (*p ≤* 0.05) among hours for each species, whereas asterisks indicate significant differences (*p ≤* 0.05) among species for each hour.

The diurnal patterns of F_v_/F_m_ were very similar in spring and autumn in *P. latifolia* and *C. incanus*, showing, in general, a decrease during the central hours of the day and a recovery in late afternoon (Fig. 5). In summer, *P. latifolia* experienced the largest diurnal variation in F_v_/F_m_, with a significant decrease from the morning (0.8) to midday (0.67) and a recovery in the afternoon (Fig. 5b). In *C. incanus*, the summer midday decrease in F_v_/F_m_ was less pronounced than that in *P. latifolia*, showing midday F_v_/F_m_ values similar to those measured in spring (0.77), without recovery in the afternoon. *P. lentiscus* had almost constant and high values of F_v_/F_m_ throughout the day during the whole growing season (Fig. 5a-c) and showed a significant midday decrease only in spring and summer (Fig. 5a, c). The daily trends of Φ_PSII_ were similar in all three species in spring, with *P. latifolia* showing significantly higher values than *C. incanus* and *P. lentiscus* only in the early morning (Fig. 5d). In summer, in contrast, there were significant differences among the three species in the daily course of Φ_PSII_, as *P. latifolia* showed the highest and *C. incanus* the lowest values, respectively (Fig. 5e). In autumn, the daily course of Φ_PSII_ in *P. lentiscus* was consistently lower than those of *C. incanus* and *P. latifolia*(Fig. 5e).

**Figure 6:**
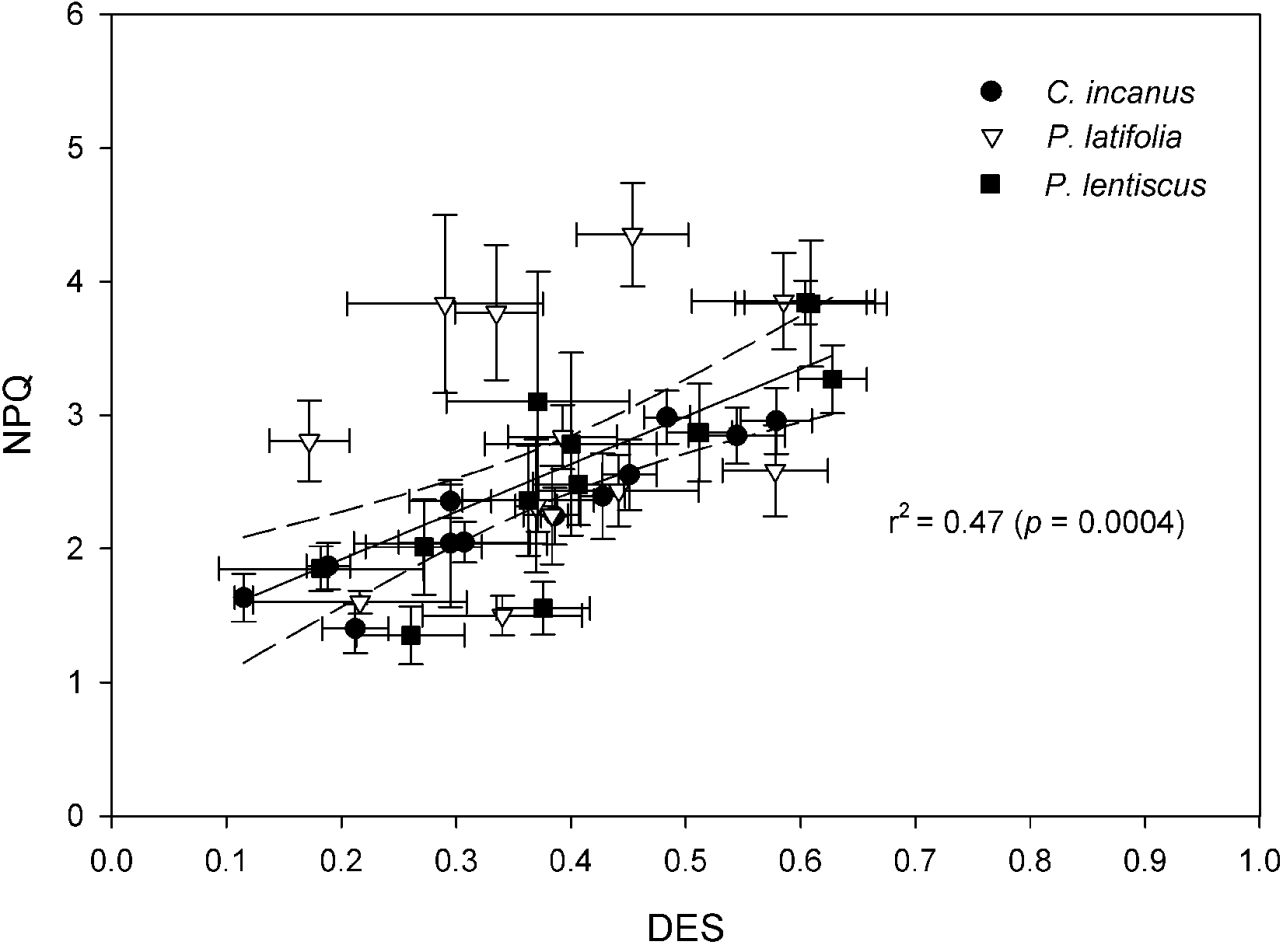
Relationship between NPQ, and de-epoxidation state of the DES in *C. incanus*, *P. latifolia* and *P. lentiscus* measured daily in spring, summer and autumn. Dara are means ± SD (n = 36). The central black line indicates the line of best-fit. The dotted lines either side of the best-fit line indicate 95% confidence intervals of the mean; *p* and *r*^2^ values indicate the results of linear regression.

The values of NPQ and DES were significantly correlated in all species (Fig. S1). In addition, the positive linear relationship between the daily values of these two parameters indicated that in all the three species, the increment in NPQ was attributable to the high de-epoxidation state of xanthophyll (Fig. 6).

**Figure 7:**
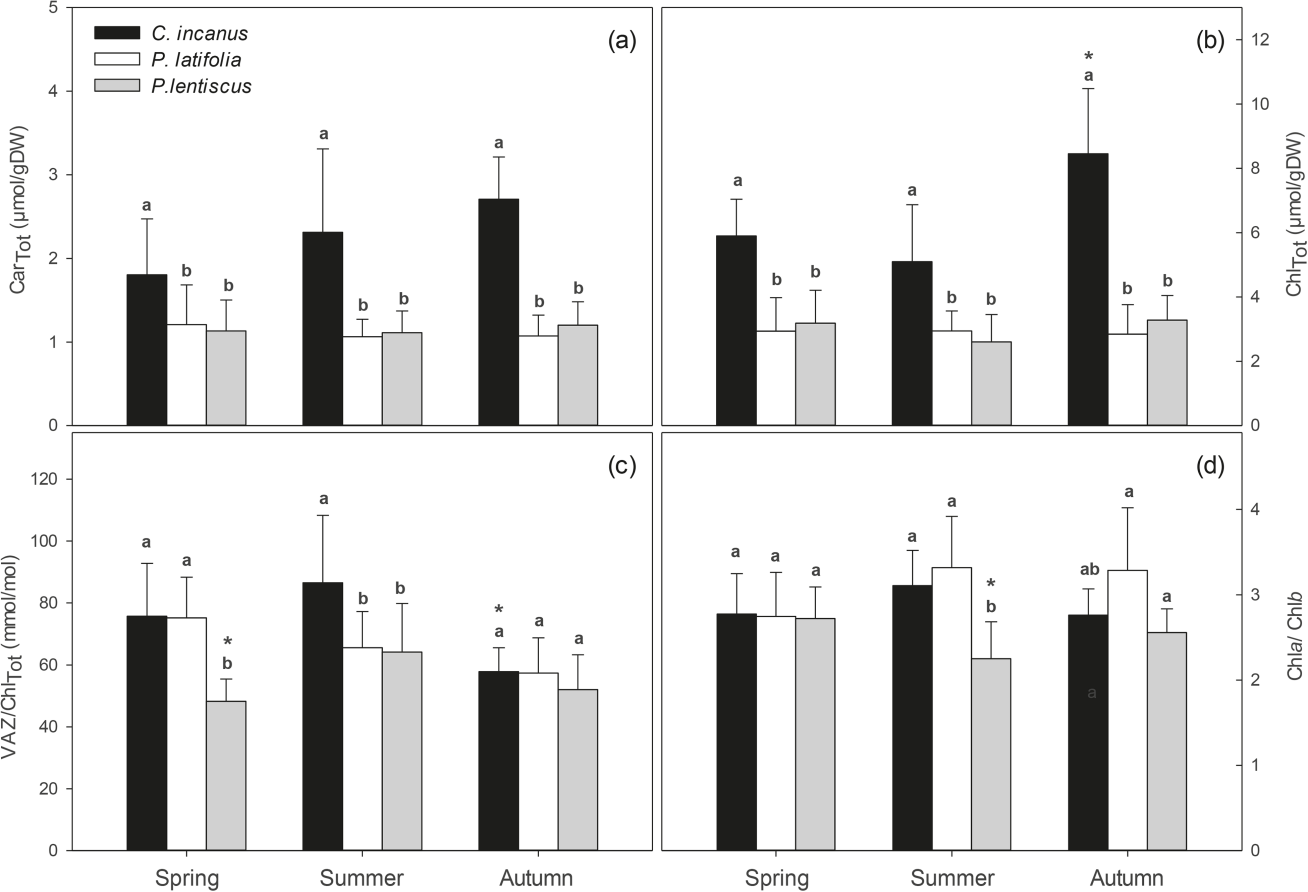
Seasonal trends in (a) total carotenoid content (Car_Tot_), (b) total chlorophyll content (Chl_Tot_), (c) xanthophyll cycle pigments to chlorophyll total ratio (VAZ/Chl_Tot_), and (d) chlorophyll *a* to chlorophyll *b* ratio (Chl*a*/Chl*b*) in *C. incanus, P. latifolia* and *P. lentiscus*. Data are means ± SD (n = 32). Letters indicate significant differences (*p ≤* 0.05) among species for each season, whereas asterisks indicate significant differences (*p ≤* 0.05) among seasons for each species.

**Figure 8:**
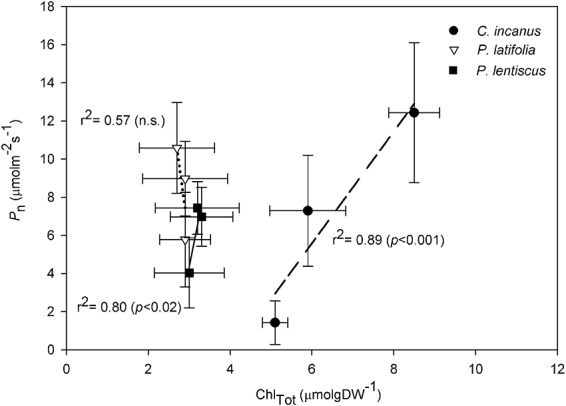
Relationship between net photosynthetic rate (*P*_n_) and total chlorophyll content (Chl_Tot_) in *C. incanus*, *P. latifolia* and *P. lentiscus* in spring, summer and autumn. Dara are means ± SD (n = 3). The lines indicate the best-fit for the three species. *P* and *r*^2^ values indicate the results of linear regression.

There were no significant seasonal changes in the content of both Car_Tot_ (Fig. 7a) and Chl_Tot_ (Fig. 7b) in *P. latiofolia* and *P. lentiscus*. In *C. incanus*, the content of Car_Tot_ and Chl_Tot_ was significantly higher, and often more than double, compared with *P. latifolia* and *P. lentiscus*. *C. incanus* also showed a significant seasonal change in Chl_Tot_, which peaked towards the end of the growing season (Fig. 7b). In addition, a strong linear relationship between Chl_Tot_ and *P*_n_ was found for this species (*r*^2^ = 0.89, Fig. 8).

Seasonality did not affect VAZ/Chl_Tot_ in *P. latifolia,* while this ratio increased significantly in autumn and declined significantly in summer and autumn in *P. lentiscus* and in *C. incanus*, respectively (Fig. 7c). In addition, in *C. incanus*, VAZ/Chl_Tot_ resulted higher than that in *P. lentiscus* in both spring and summer, while it was significantly higher than *P. latifolia* only in summer (Fig. 7c). Finally, *P. lentiscus* had a significant decrease in Chl*a*/Chl*b* in summer (Fig. 7d).

**Table 1:** *P* values from two-way repeated-measures analysis of variance (RP-ANOVA) and *η*^2^ (eta-squared value) for the effects of ‘species’, ‘season’ and ‘sampling hour’ and their interaction on physiological (net photosynthesis, *P*_n_; stomatal conductance, *g*_s_; leaf water potential, *Ψ*_w_; leaf osmotic potential, *Ψ*_π_; maximum photochemical efficiency of PSII, F_v_/F_m_; actual efficiency of PSII, Φ_PSII_; NPQ) and biochemical traits (DES (antheraxanthin + zeaxanthin) (antheraxanthin + zeaxanthin + violaxanthin)^−1^; Car_Tot_, content of total carotenoids; Chl_Tot_, content of total chlorophylls; Chl*a*/Chl*b*, chlorophyll a: chlorophyll b ratio; VAZ/Chl_tot_, xanthophyll cycle pigments to chlorophyll total ratio; Pol_Tot,_ concentration of total polyphenols; PP_Tot_, concentration of total phenylpropanoids; )

	*P*_n_		*g*_s_		*Ψ*_w_		*Ψ*_π_		F_v_/F_m_		Φ_PSII_		NPQ		DES	
Sources	*p*	*η* ^2^	*p*	*η* ^2^	*p*	*η* ^2^	*p*	*η* ^2^	*p*	*η* ^2^	*p*	*η* ^2^	*p*	*η* ^2^	*p*	*η* ^2^
Species	<0.001	0.087	<0.001	0.086	<0.001	0.248	<0.001	0.461	<0.001	0.095	<0.001	0.082	<0.001	0.094	<0.001	0.097
Season	<0.001	0.510	<0.001	0.245	<0.001	0.274	<0.001	0.134	<0.001	0.184	<0.001	0.074	<0.001	0.243	<0.001	0.100
Sampling hour	<0.001	0.188	<0.001	0.189	<0.001	0.240	<0.001	0.126	0.003	0.515	<0.001	0.643	0.002	0.453	<0.001	0.578
Species x Season	<0.001	0.172	<0.001	0.424	<0.001	0.166	<0.001	0.179	<0.001	0.093	0.007	0.068	<0.001	0.071	<0.001	0.088
Species x Sampling hour	<0.001	0.043	<0.001	0.056	<0.001	0.072	<0.001	0.100	0.005	0.114	<0.001	0.132	0.005	0.139	0.009	0.137
	Car_Tot_		Chl_Tot_		VAZ/Chl_Tot_		Chl*a*/Chl*b*		Pol_Tot_		PP_Tot_		ABA		ABA-GE	
Sources	*p*	*η* ^2^	*p*	*η* ^2^	*p*	*η* ^2^	*p*	*η* ^2^	*p*	*η* ^2^	*p*	*η* ^2^	*p*	*η* ^2^	*p*	*η* ^2^
Species	<0.001	0.611	<0.001	0.052	<0.001	0.281	<0.001	0.253	<0.001	0.464	<0.001	0.243	<0.001	0.403	<0.001	0.683
Month	<0.001	0.100	<0.001	0.443	<0.001	0.239	<0.001	0.193	<0.001	0.180	<0.001	0.230	<0.001	0.258	<0.001	0.100
Sampling hour	<0.001	0.088	<0.001	0.396	<0.001	0.198	0.253	0.136	<0.001	0.079	<0.001	0.043	<0.001	0.086	<0.001	0.043
Species x Season	<0.001	0.141	<0.001	0.068	<0.001	0.198	<0.001	0.222	<0.001	0.208	<0.001	0.447	<0.001	0.142	<0.001	0.120
Species x Sampling hour	<0.001	0.059	<0.001	0.041	0.450	0.084	<0.001	0.197	<0.001	0.068	<0.001	0.037	<0.001	0.121	<0.001	0.054

**Figure 9:**
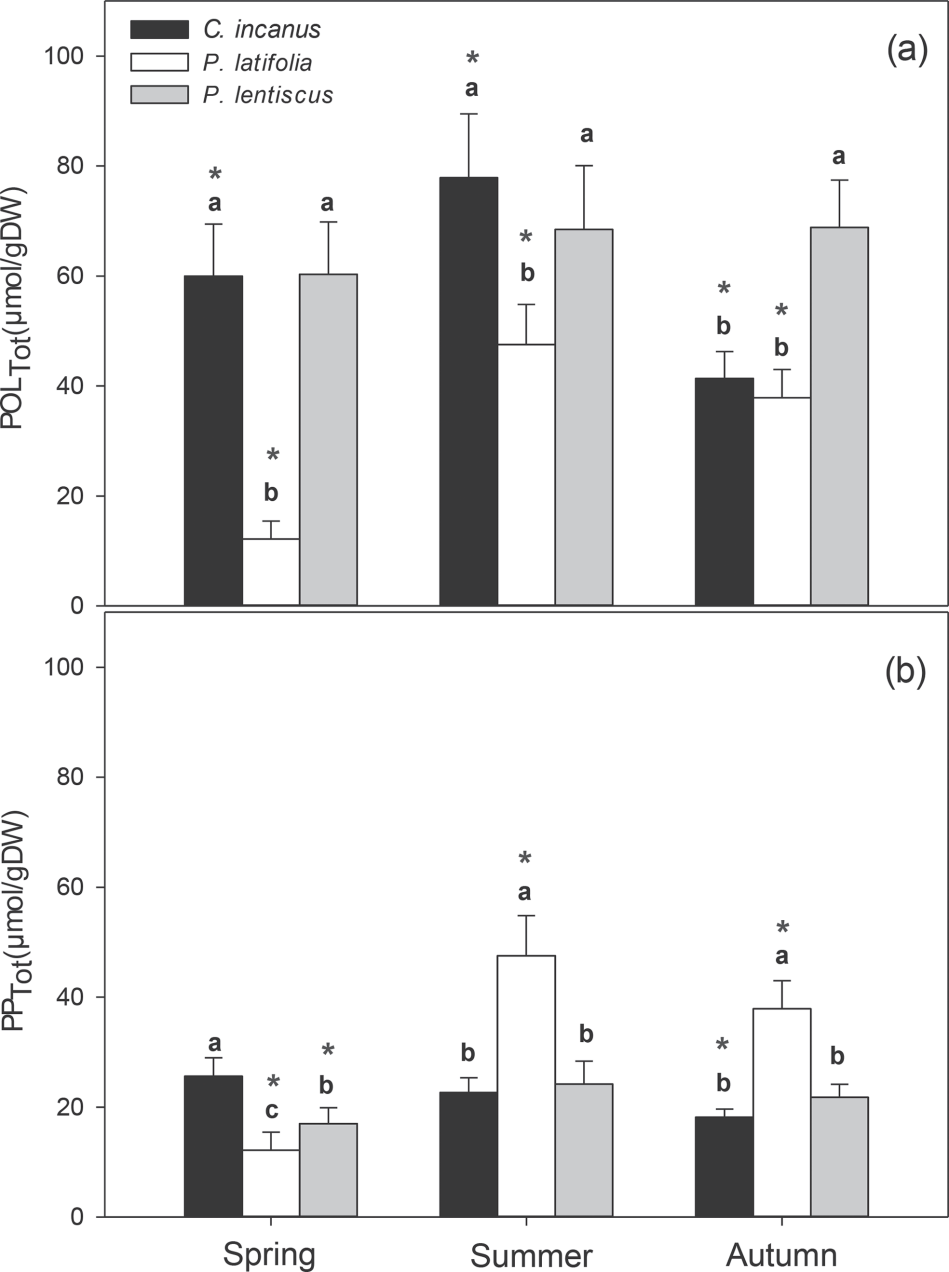
Seasonal trends in content of (a) total polyphenols (POL_Tot_) and (b) total phenylpropanoids (PP_Tot_) in *C. incanus, P. latifolia* and *P. lentiscus*. Data are means ± SD (n = 32). Different lower-case letters indicate significant differences (*p ≤* 0.05) among species for each season, whereas asterisks indicate significant differences (*p ≤* 0.05) among seasons for each species.

The analyses of polyphenol content showed that POL_Tot_ increased significantly in summer both in *C. incanus* and in *P. latifolia* but not in *P. lentiscus*, as this latter species maintained constant levels of POL_Tot_ throughout the whole growing season (Fig. 9a). *P. latifolia* formed only phenylpropanoids and, consequently, POL_Tot_ resulted significantly lower than in the other two species in all seasons. In *C. incanus*, POL_Tot_ declined sharply in autumn and resulted significantly lower than that in *P. lentiscus*. The PP_Tot_ seasonal trend differed significantly among the three species (Fig. 9b). In fact, while PP_Tot_ significantly increased in *P. latifolia* and to a lesser extent in *P. lentiscus* in summer and autumn, *C. incanus* showed a decline in PP_Tot_ in autumn. Furthermore, while in spring the PP_Tot_ in *P. latifolia* was noticeably lower than that in *P. lentiscus* and less than half of the content in *C. incanus*, as the growing season progressed, PP_Tot_ was dramatically stimulated in *P. latifolia* and became approximately double than that of the content in the other two species.

**Figure 10:**
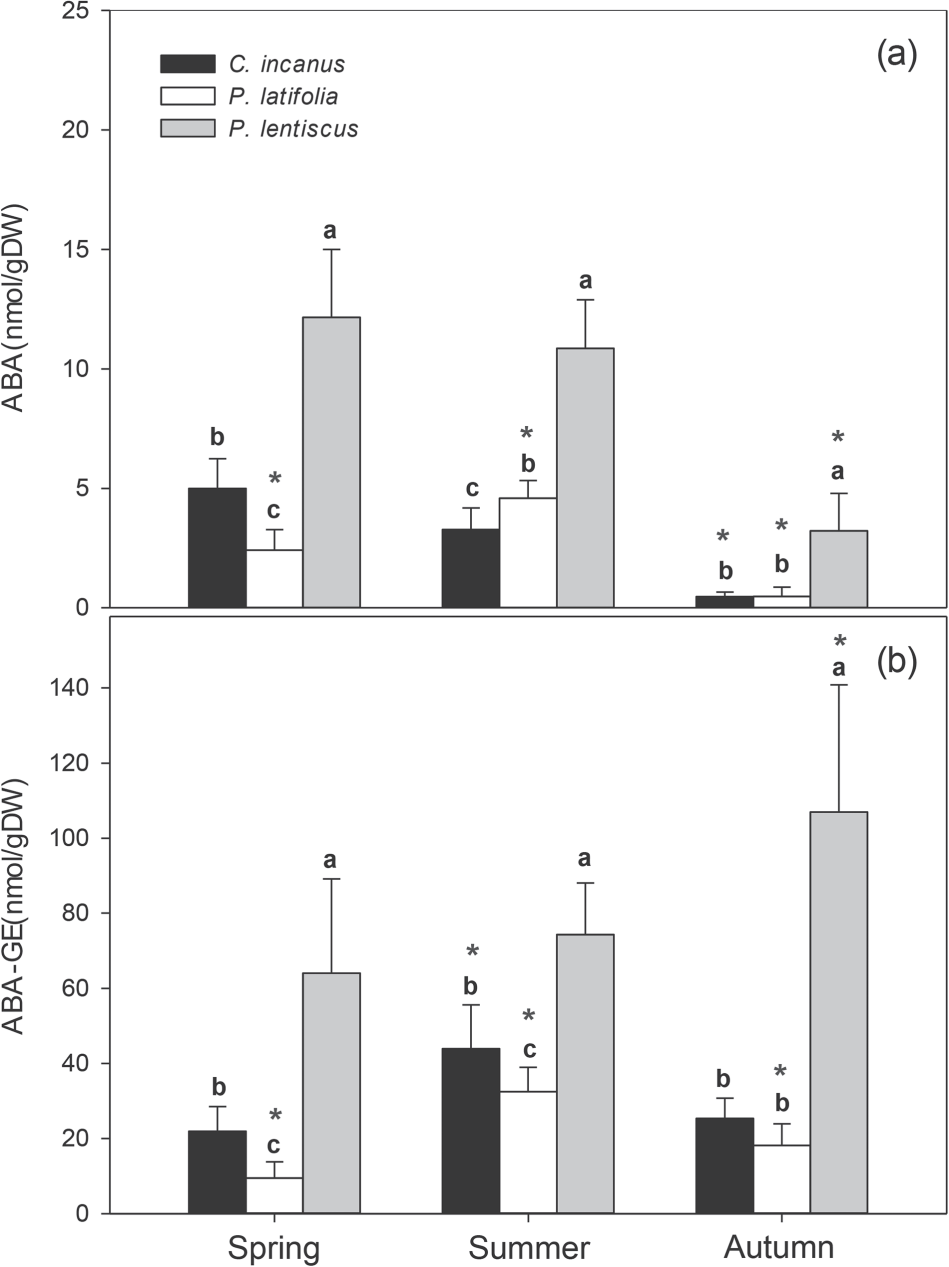
Seasonal variation in content of (a) ABA and (b) ABA-GE in *C. incanus, P. latifolia* and *P. lentiscus*. Data are means ± SD (n = 32). Letters indicate significant differences (*P ≤* 0.05) among species for each season, whereas asterisks indicate significant differences (*p ≤* 0.05) among seasons for each species.

The seasonal trends in leaf ABA (Fig. 10a) and ABA-GE (Fig. 10b) differed significantly among the three species. In *P. latifolia*, ABA and ABA-GE contents had a similar seasonal course, increasing significantly from spring to summer to then decrease towards the end of the growing season. In *C. incanus* and in *P. lentiscus*, free ABA became dramatically lower in autumn, while ABA-GE increased significantly in summer (+90%) and in autumn (+41%) in *C. incanus* and *P. lentiscus*, respectively. *P. lentiscus* was characterized by much higher ABA and ABA-GE contents (up to 10-fold higher in free-ABA) than in the other two species during the whole growing season. In this species, the spring values of both ABA and ABA-GE were around 12 nmol g^−1^ DW^−1^ and 64 nmol g^−1^ DW^−1^, respectively. These levels were maintained constant in summer, while in autumn, free ABA decreased significantly (~3 nmol g^−1^ DW^−1^) concomitantly with a sharp increase in ABA-GE (~107 nmol g^−1^ DW^−1^). There were significant differences in ABA contents also between *C. incanus* and *P. latifolia*, although there was no clear seasonal trend, whereas ABA-GE resulted higher in *C. incanus* than *P. latifolia* both in spring and summer and became similar in autumn.

### Whole trait relationship (PCA)

**Figure 11:**
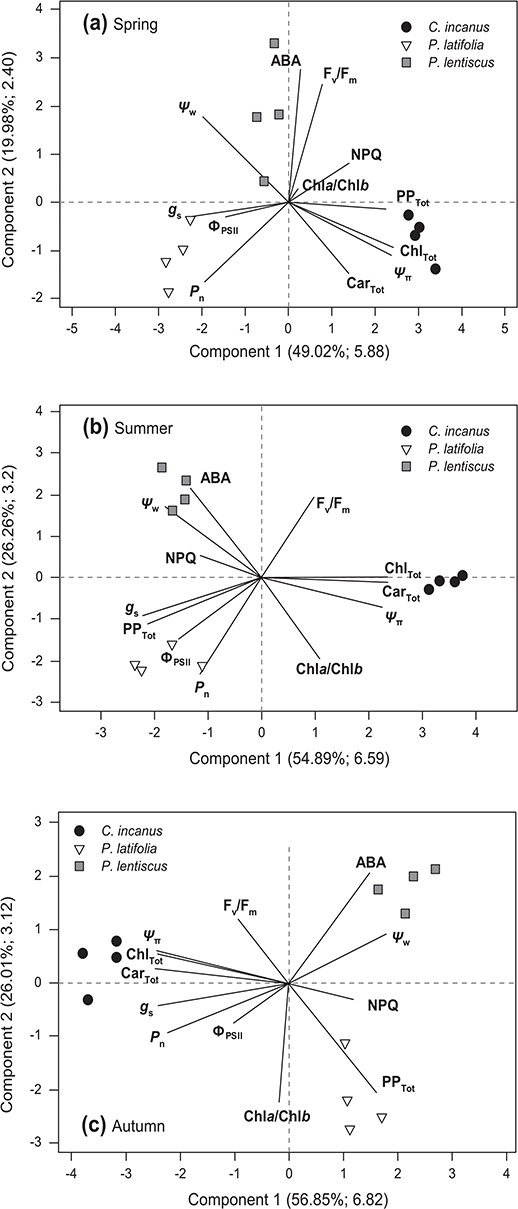
PCA performed using physiological traits ( *P*_n_, *g*_s_, Ψ_w_, Ψ_π_, Φ_PSII_, NPQ and Fv/F_m_,) and biochemical traits (PP_Tot_, Car_Tot_, Chl_Tot_ Chl*a*/Chl*b* and ABA) in spring (a) summer (b) and autumn (c) for the considered species. In the parentheses it is shown the percentage of total variation explained by each PC axis and the relative eigenvalues.

PCA shows the relationships among all traits studied. In particular, PCA shows that the three species differ in their placement within the trait-space during the whole growing season (Fig. 11). In spring (Fig. 11a), the first component of PCA, which accounted for 49% of the total variance, was defined by the opposition between *C. incanus* on the positive side (associated with Car_Tot_, Chl_TOt_, PP_Tot_ and *Ψ*_π_) and *P. latifolia* (associated with *P*_n_, *g*_s_, and Φ_PSII_) on the negative side. The second component, accounting for 19.9% of the total variance, opposed *P. lentiscus* (characterised by positive, high values of ABA and F_v_/F_m_) and the other two species. In summer (Fig. 11b), the first and the second principal components explained the 54.9% and 26.3% of the total variance, respectively. *C. incanus* was highly associated with component one and was characterised by a high content of Car_Tot_ and Chl_Tot_ and low values of *Ψ*_π_. The second component divided the evergreens, with *P. lentiscus* on the positive side, characterized by high values of *Ψ*_w_ and ABA and low values of Chl*a*/Chl*b*, and *P. latifolia* on the negative side, associated with high *P*_n_, *g*_s_, Φ_PSII_ and PP_Tot_ and low F_v_/F_m_ values. Finally, in autumn (Fig. 11c), the first axis (56.9% of the total variance) divided *C. incanus* from the evergreens, whereas the second axis (26% of the total variance) separated *P. lentiscus* (positive scores) from *P. latifolia* (negative scores)*. P. latifolia* was characterized by PP_Tot,_*C. incanus* by *g*_s_, *P*_n_, Car_Tot_, Chl_Tot_ and *Ψ*_π,_ and *P. lentiscus* by *Ψ*_w_ and ABA.

### Influence of climatic factors on physiological and biochemical traits

The effects of the climatic factors (temperature, global irradiance and precipitation) on the physiological and biochemical traits of the study species were assessed through MRA (Tab. 2). In general, all relationships among meteorological data and physiological and biochemical parameters were highly significant in all species (*p* < 0.05), with the exception of *g*_s_ in *P. latifolia*. MRA showed a strong influence of the climatic factors on water relation parameters, especially on *Ψ*_w_ in *P. latifolia* (*r*^2^ = 0.75) and in *C. incanus* (*r*^2^ = 0.70). In these two species, a strong negative relationship was found between *Ψ*_w_ with precipitation (P). Conversely, the relationships between *P*_n_ and *g*_s_ and the climate variables were generally weak, with higher *r*^2^ values for *P*_n_ in *C. incanus* (*r*^2^ = 0.47) and *P. lenticus* (*r*^2^ = 0.54) than in *P. latifolia* (*r*^2^ = 0.32). Temperature (T) was the climatic factor which mostly contributed to *P*_n_ reductions in all species. The relationship between F_v_/F_m_ with climatic factors strongly differed among species, with higher *r*^2^ values in *C. incanus* (*r*^2^ = 0.65) and in *P. latifolia* (*r*^2^ = 0.52) than in *P. lentiscus* (*r*^2^ = 0.37). In contrast, climatic factors did not strongly affect Φ_PSII_, irrespective of the species (*r*^2^ was on average ~ 0.35). Parameters linked to the thermal dissipation of excess energy, such as NPQ and DES, were strongly and positively correlated to air temperature in the examined species. Consistently, strong correlations between climatic variables and photosynthetic pigments were found, with higher *r*^2^ values for *C. incanus* than the other two species. In particular, temperature negatively affected Chl_Tot_ (*r*^2^ = 0.86) and increased VAZ/Chl_Tot_ ratio (*r*^2^ = 0.6) in *C. incanus*. In addition, temperature decreased Chl*a*/Chl*b* in *P. lentiscus* (*r*^2^ = 0.52). Finally, temperature had a positive influence on polyphenol content in all species, and especially in *P. latifolia*, in which T was correlated with PP_Tot_. In this species, a strong relationship between T and ABA (*r*^2^ = 0.54) was also found.

**Table 2:** MRA showing the influence of air temperature (T, °C), global irradiance (GI, Wm^−2^) and precipitation (P, mm) on physiological (net photosynthesis, *P*_n_; stomatal conductance, *g*_s_; leaf water potential, *Ψ*_w_; leaf osmotic potential, *Ψ*_π_; maximum photochemical efficiency of PSII, F_v_/F_m_; actual efficiency of PSII, Φ_PSII_; NPQ), and biochemical traits (DES (antheraxanthin + zeaxanthin) (antheraxanthin + zeaxanthin + violaxanthin)^−1^; Car_Tot_, content of total carotenoids; Chl_Tot_, content of total chlorophylls; Chl*a*/Chl*b*, chlorophyll a: chlorophyll b ratio; VAZ/Chl_tot_, xanthophyll cycle pigments to chlorophyll total ratio; Pol_Tot,_ concentration of total polyphenols; PP_Tot_, concentration of total phenylpropanoids; ) of the study species

	*P. latifolia*				*C. incanus*				*P. lentiscus*			
Parameter	*r* ^2^	T (°C)	GI (Wm^−2^)	P (mm)	*r* ^2^	T (°C)	GI (Wm^−2^)	P (mm)	*r* ^2^	T (°C)	GI (Wm^−2^)	P (mm)
*Ψ*_w_	0.75^***^	−0.038	−0.001	−0.187	0.70^***^	−0.020	−0.001	−0.194	0.47^***^	−0.009	−0.001	−0.119
*Ψ*_π_	0.38^***^	−0.022	−0.001	−0.092	0.67^***^	−0.012	0.000	−0.127	0.41^***^	−0.007	0.000	−0.018
*P*_n_	0.32^***^	−0.480	0.003	−0.008	0.47^***^	−0.851	0.002	−0.031	0.54^***^	−0.481	0.003	−0.001
*g*_s_	0.03 ^n.s.^	−0.060	−0.015	−0.079	0.25^***^	−0.705	−0.010	−0.385	0.23^***^	−0.813	0.014	−0.249
F_v_/F_m_	0.52^***^	−0.018	−0.009	0.000	0.65^***^	−0.012	−0.007	0.000	0.37^***^	−0.001	−0.004	0.000
Φ_PSII_	0.35^***^	0.000	0.000	−0.001	0.31^***^	−0.005	0.000	0.000	0.39^***^	−0.001	0.000	−0.001
NPQ	0.78^***^	0.195	0.004	0.001	0.67^***^	0.053	0.003	0.000	0.79^***^	0.120	0.002	0.009
DES	0.73^***^	0.020	0.001	0.000	0.83^***^	0.012	0.001	0.000	0.59^***^	0.017	0.000	0.000
Car_Tot_	0.30^***^	0.015	0.001	0.001	0.51^***^	0.013	0.000	0.002	0.23^***^	0.004	0.000	0.003
Chl_Tot_	0.30^***^	0.005	−0.001	0.007	0.86^***^	−0.162	−0.025	0.033	0.16^***^	−0.003	−0.001	0.007
Chl*a*/Chl*b*	0.11^*^	0.049	0.000	−0.001	0.48^***^	0.054	−0.001	−0.004	0.52^***^	−0.039	0.020	0.001
VAZ/Chl_tot_	0.41^***^	0.770	0.014	−0.170	0.61^***^	1.979	0.032	−0.086	0.39^***^	1.821	0.011	−0.064
Pol_Tot_	0.68^***^	3.643	0.203	0.050	0.27^***^	1.103	0.030	−0.039	0.13^**^	0.676	0.000	0.083
PP_Tot_	0.68^***^	3.643	0.203	0.050	0.25^***^	0.429	0.007	−0.032	0.27^***^	0.471	−0.001	0.048
ABA	0.54^***^	0.226	0.001	0.014	0.25^***^	0.136	0.000	0.028	0.23^***^	1.000	0.000	0.070

^***^ indicates significance difference at *p* ≤ 0.001,

^**^ indicates significance difference at *p* < 0.01,

^*^ indicates significance difference at *p* < 0.05, n.s. indicate no significant differences.

## Discussion

In this study we used a trait-based approach to explore how coexisting woody shrubs responded to Mediterranean climate in their natural environment. Trait-based studies might be particularly relevant in the near future, leading to a better understanding of the role of plant traits on community dynamics of Mediterranean ecosystems, especially in the context of climate change (Lloret *et al.*, 2013). Indeed, the different responses of coexisting species to extreme climate events and their relationships with key functional traits remain poorly understood and may help to increase the comprehension of the ecophysiological mechanisms involved in plant vulnerability and resilience (Matusick *et al.*, 2012; McDowell, 2011).

We found large differences in primary and secondary metabolism among the three Mediterranean maquis species. Contrasting behaviours were especially observed on a seasonal level (Tab. 1, Fig. 12), while MRA revealed that air temperature had a stronger effect than precipitation and irradiance in determining the range of variation in several traits related to the physiology and the biochemistry of the maquis shrubs of coastal dunes (Tab. 2). The Mediterranean climate is characterized by a strong seasonality, e.g. mild springs, with increasing temperatures mirrored by progressively declining soil water content, followed by hot and dry summers and then by autumn with mild temperatures accompanied by abundant rainfalls (Barbero *et al*., 1992). Thus, the temperature trend is a strong indicator of the incoming season and the associated stress conditions (e.g. long harsh summers characterized by high temperatures, heat waves and concomitant droughts) (Barriopedro *et al*., 2011) and, therefore, is a major driver of multifunctionality in areas characterised by strong environmental stresses (Jing *et al*., 2015; Maestre *et al*., 2012).

In *P. latifolia*, the large differences between *Ψ*_wMD_ and *Ψ*_wPD_ (Δ*Ψ*_w_) (Tab. 3) and the strict correlation between *g*_s_ and *Ψ*_w_ (Fig. S1) over the whole growing season, demonstrated a typical anisohydric behaviour, as previously observed by other authors (Peñuelas *et al*., 1998; Ogaya and Peñuelas, 2003; Gratani *et al*., 2013). This species is classified as drought-tolerant because of its ability to adjust osmotic potential under water deficit conditions (Borghetti *et al*., 2004; Tattini *et al*., 2002; Mereu *et al*., 2009; Liu *et al*., 2011). Accordingly, in this species, we observed the largest Δ*Ψ*_π_ and the lowest values of *Ψ*_πPD_ and *Ψ*_πMD_ in summer (Table 3, Fig. S2). The reduction in osmotic potential allows leaf cell turgor to be maintained through the active accumulation of solutes, thus facilitating the extraction of water from dried soils and permitting the maintenance of relatively high gas exchanges in summer under high temperature and low precipitation (Fig. 2, Table 2) (Kramer and Boyer, 1995). This could be also confirmed by the significant relationship found between WUEi and *Ψ*_w_, indicating that this species presented a tight control on stomatal conductance under drought conditions, resulting in a high efficiency in terms of water use (Fig. 4).

**Table 3:** Gradient between predawn and midday water (*ΔΨw*) and osmotic (*ΔΨπ*) potential measured in spring, summer and autumn in *C. incanus*, *P. latifolia* and *P. lentiscus*. Data are means of 8 plants ± SD. Letters indicate significant differences (*p ≤* 0.05) within the same species in different seasons, whereas asterisks indicate significant differences (*p ≤* 0.05) among species

	***C. incanus***	***P. latifolia***	***P. lentiscus***	
***ΔΨw***	1.12 ± 0.20 a^*^	1.51 ± 0.37 a^*^	0.46 ± 0.05 a^*^	Spring
	0.99 ± 0.19 a^*^	2.72 ± 0.73 b^*^	0.45 ± 0.06 a^*^	Summer
	0.81 ± 0.18 a^*^	1.28 ± 0.17 a^*^	0.44 ± 0.10 a^*^	Autumn
***ΔΨπ***	0.35 ± 0.12 a	0.42 ± 0.17 a	0.16 ± 0.07 a^*^	Spring
	0.48 ± 0.13 a	0.81 ± 0.23 b^*^	0.24 ± 0.13 b	Summer
	0.30 ± 0.10 a^*^	0.57 ± 0.13 a^*^	0.18 ± 0.09 a^*^	Autumn

Similarly, *C. incanus,* as an anisohydric species, showed large variations in Δ*Ψ*_w_ during the whole growing season (Tab. 3) and a highly significant negative relationship between *g*_sMD_ and *Ψ*_wMD_ (Fig. 4a). As already reported, *Cistus* spp. behave as drought-avoider water-saver plants, showing partial leaf-shedding during summer, combined with a decrease in *g*_s_ and no active accumulation of osmolytes in the retained leaves (Werner *et al*., 1999; Sánchez-Blanco *et al*., 2002; Bombelli and Gratani, 2003). Consistently, *C. incanus* had a high seasonal variability in gas exchanges and water potential (Fig. 2, Fig. S2) and this could be explained by its shallow root system that allows it to respond fast to the first autumn rainfalls but renders it more sensitive to water stress (Gallé *et al*., 2011; Correia *et al*., 2014) as well as due to its ability to diachronically shift leaf-level strategies in the medium-term (Correia and Ascensão, 2017; Puglielli *et al*., 2017a; Puglielli *et al*., 2017b). Therefore, both temperature and precipitation had a great influence on *P_n_* and *Ψ*_w_ in this species (Tab. 2).

Finally, *P. lentiscus* displayed nearly constant daily *Ψ*_w_ and *g*_s_ during the whole growing season (Fig. S2, Fig. 2). For this species, the relationship between these two parameters was not significant (Fig. 4a). In this plant, stomata remain open during the summer *Ψ*_wMD_ reduction which implies high water consumption and consequently low WUEi (Fig. 4b). This physiological homeostasis is typical of a drought-avoider water-spender plant and could be related to the capacity of this species to extract water from soil rapidly enough to compensate water loss by transpiration (Armas *et al*., 2010; Ozturk *et al*., 2010). Accordingly, water relations were only partially related to changes in precipitations (Tab. 2). This supports previous experiments utilizing isotopic abundance analysis (with *δ*^18^O and *δ*^13^C), which provided evidence that the deep root system of *P. lentiscus* allows the maintenance of a favourable plant water supply even under severe drought (Ehleringer and Dawson, 1992; Valentini *et al*., 1992; Filella and Peñuelas, 2003). However, in the present work, the photosynthetic rates found for the species were slightly lower than those reported in previous studies (Gulías *et al*., 2009).

The divergent physiology of the three shrubs is also highlighted by their different daily patterns of maximal and actual PSII efficiency (Fig. 5). The drought-tolerant *P. latifolia* was apparently the most affected by the severe environmental conditions of the Mediterranean summer. This species showed a daily significant reduction in F_v_/F_m_, and this was probably due to the complementary action of high temperature and high irradiance (Fig. 5b, Tab. 2). However, the F_v_/F_m_ ratio recovered rapidly late in the afternoon (18:00 h), indicating that there was no damage to the reaction centres. This could indicate a process of dynamic photoinhibition in the photosynthetic apparatus, in which the drop in F_v_/F_m_ over the course of the day operated in tandem with the thermal dissipation activity (NPQ) associated with DES without impairment of PSII. This is consistent with the significant correlation between NPQ and DES in all species (Fig. 6) (Demmig-Adams and Adams, 1996; Kyparissis *et al*.*,* 2000; Martínez-Ferri *et al*., 2000). Therefore, in *P. latifolia*, we only found a significant but slight downregulation of Φ_PSII,_ which was significantly higher compared to the other two species during the central hours of the day (Fig. 5e).

*C. incanus* and *P. lentiscus* appeared to be less susceptible to summer photoinhibition than *P. latifolia,* as only a slight summer decrease in F_v_/F_m_ was observed at midday for both species (Fig. 5 a-c). Photoinhibition avoidance in *Cistus* spp. and *P. lentiscus* has previously been described by other authors (Oliveira and Peñuelas, 2000; Werner *et al*., 2002; Ain-Lhout *et al*., 2004; Valladares and Sánchez-Gómez, 2006) and could be attributable to their peculiar morphological features (leaf pubescence and vertical orientation for *C. incanus* and epicuticular waxes for *P. lentiscus*) that may effectively contribute to increasing leaf surface reflectance and reducing photon absorbance (Núñez-Olivera *et al*., 1996; Rossi *et al*., 2001). However, the daily recovery of the maximum efficiency of PSII was slower in the semi-deciduous species in comparison to the two evergreens, as *C. incanus* reached optimum F_v_/F_m_ values only in the morning (Fig. 5b). In addition, recent evidences have shown that, for *Cistus* spp., the light harvesting complex structure can change in leaves developed under seasonally different environmental conditions, thus leading to changes in F_v_/F_m_ (Grant *et al*., 2015; Puglielli *et al.,* 2017b).

Our analysis of seasonal dynamics in plant photoprotective pigments revealed similarities and differences among the three species. The slight and non-significant differences in photosynthetic pigments observed in *P. latifolia* through the growing season (Fig. 7a) and the low impact of climatic factors on these biochemical traits (Tab. 2) suggest that, in this Mediterranean evergreen, xanthophylls cycle pigments and leaf chlorophylls were mainly adjusted according to the need to dissipate excess of excitation energy rather than following seasonal variations in both temperature and irradiance (Kyparissis *et al*., 2000; Gratani *et al*., 2006). On the contrary, the seasonal modulations of chlorophyll contents observed in *C. incanus*, with significant lower levels in summer (Fig. 7b), suggest for this species mechanisms of adaptation to high irradiance and high temperatures (Tab. 2) (Hernández *et al*., 2004; Grant *et al*., 2015). This chlorophyll loss in *C. incanus* induced by high summer temperatures (Tab. 2) could have led to an increased VAZ/Chl_Tot_ ratio (Fig. 7c), thus enhancing the capacity to dissipate excess excitation energy per amount of light intercepted and limit lipid peroxidation (Galmés *et al*., 2007; Munné-Bosch *et al*., 2009). In addition, the larger carotenoid and chlorophyll contents found in the semi-deciduous compared to the two evergreens could have allowed the maintenance of high rates of photosynthesis under well-watered conditions (Correia *et al*., 2014) (Fig. 8). Consistently, a positive relationship between Chl_tot_ and precipitation was found for this species (Tab. 2), with the highest values of *P*_n_ recorded after the first rainfalls in autumn (Fig. 3a). In the other evergreen, *P. lentiscus*, a seasonal modulation in the Chl*a*/Chl*b* ratio was observed (Fig. 7d). In this species, the summer reduction in Chl*a* may lead to changes in PSII/PSI balance, thus offering a protective mechanism against potentially damaging effects caused by high irradiance and high temperature (Table 2) (Munné-Bosch and Peñuelas, 2003; Vasques *et al*., 2016).

Mediterranean shrubs generally accumulate large amounts of polyphenols (Di Ferdinando *et al*., 2014). Among polyphenols, phenylpropanoids and particularly flavonoids with a catechol group in the B-ring, such as quercetin and luteolin derivatives, are among the most effective antioxidant compounds and, hence, have been reported to increase under UV radiation (Bernal *et al*., 2013; Agati *et al*., 2012). In *P. latifolia*, the observed seasonal variations in phenylpropanoid compounds may likely be related to the occurrence of abiotic stresses and, in particular, the summer increment in total phenylpropanoids might have reflected a higher need for antioxidant activity because of plant exposure to high temperatures (Fig. 9b, Table 2), as previously reported by Bautista *et al*. (2016) for other Mediterranean wild species. Conversely, in *P. lentiscus* and *C. incanus*, variations in the leaf content of polyphenols and phenylpropanoids did not show a clear seasonal trend and were weakly correlated with climatic factors (Fig. 9, Table 2), suggesting, for these compounds, different ecological functions rather than antioxidant and UV screening effects. In *P. lentiscus* and *C. incanus*, the main fraction of polyphenolic compounds is represented by tannins (Gori *et al*., 2016; Rodríguez-Pérez *et al*., 2013). Previous studies have shown that in *Cistus* spp. tannins are located in the trichome channels and, when released to the soil, may contribute to nitrogen- cycling processes (Castells *et al*., 2004; Di Ferdinando *et al*., 2014). Whereas, in *P. lentiscus*, tannins are distributed through the whole-leaf tissues, helping to strengthen the cell walls and increasing sclerophyllity (Bussotti *et al*., 1998). However, as other biotic stresses have similar effects, we cannot exclude that these species may have experienced pathogen and insect attacks, which may have contributed to the observed seasonal variation of leaf polyphenolic contents during our study (Liakoura, *et al*., 2001).

To our knowledge, little is known about leaf variations in ABA contents in Mediterranean plants exposed to a combination of abiotic stressors in natural field conditions (López-Carbonell *et al*., 2009). In our investigation, the three species presented significantly different leaf levels of free-ABA and of glucose-conjugated ABA (ABA-GE) during the whole growing season (Fig. 10a, b). However, it is already known that under drought field conditions, levels of leaf ABA increase (Rodrigues *et al*., 2008; López-Carbonell *et al*., 2009), and ABA acts as a signal of soil drying to induce stomatal closure (Zhang *et al*., 1987). However, some evidence has proposed that the initial stomatal closure is induced by a hydraulic signal followed by an increase in ABA content in droughted leaves (Christmann *et al*., 2007; Li *et al*., 2010; Speirs *et al*., 2013; Marino *et al*., 2017). In *P. latifolia*, leaf ABA content followed the same seasonal pattern of *Ψ*_w_ (Fig. 10a and Fig. S2), leading us to hypothesize a possible combination of chemical and hydraulic messages in stomatal regulation of this plant (Pantin *et al*., 2012). In addition, the observed strong relationship of leaf ABA contents with temperature may suggest the involvement of this hormone in the stomatal functioning and in the regulation of transpiration during the hot summer season (Tab. 2). This mechanism did not work for *C. incanus* and *P. lentiscus*, as climatic factors apparently had little influence on seasonal ABA variations observed in both species (Table 2, Fig. 10a). We noticed a higher leaf ABA and ABA-GE content in the isohydric *P. lentiscus* compared with the other two anisohydric plants. These results are in line with previous research and confirm a prominent role played by ABA in the responses of isohydric plants to severe seasonal drought (Soar *et al*., 2006; Lovisolo *et al*., 2008; Nolan *et al*., 2017). This hypothesis is also reinforced by the fact that the decrease in ABA levels in autumn coincides with the increase in ABA-GE in *P. lentiscus*, suggesting a modulation of ABA metabolism through the conjugation of free-ABA with glucose and the accumulation of this storage form in well-watered leaves (Lee *et al*., 2006; López-Carbonell *et al*., 2009; Zarrouk *et al*., 2016). Therefore, divergent climatic factors and water-use behaviours were found to be associated with different seasonal patterns of ABA among species. Therefore, changes in the content of this hormone may help to optimize the physiological performances of these plants in their natural habitat.

To summarize, our study showed that Mediterranean coexisting shrubs strongly differ in their physiological and biochemical responses and show contrasting behaviours especially on a seasonal level (Fig. 11). In *P. latifolia*, as shown by PCA analyses, the drought-tolerant behavior combined with a great investment in phenylpropanoids allowed the maintenance of actual efficiency of PSII, resulting in high photosynthetic rates through the whole growing season. The semi-deciduous *C. incanus* had the highest amounts of carotenoids and the capacity to adjust chlorophyll content on a seasonal timescale. These mechanisms help protect the efficiency of PSII in spring and summer, and, at the same time, contribute to the recovery of photosynthetic capacity after the first rainfalls in autumn.

Finally, in the isohydric *P. lentiscus*, the elevated levels of ABA allowed a strict control of stomata throughout the growing season, while the fine regulation of Chl*a*/Chl*b* concurred to avoid photoinhibition in summer.

Although water availability is considered one of the most important factors in semi-arid ecosystems (Hoerling *et al*., 2012), our results suggest that the increase in air temperature predicted by climate change projections may impose major constrains to Mediterranean maquis shrubs. Indeed, several authors have already reported that both chronic and abrupt heat stress may impact plants not only through direct effects on physiological performances, as we have tested in our field experiment, but also through indirect processes such as altering phenological processes (Hatfield and Prueger, 2015) and limit nutrient availability (Bond-Lamberty and Thomson, 2010). Moreover, the effects of high temperature and water deficit stress, both of which characterize semi-arid ecosystems, are globally additive and their combined effect is known to be even more deleterious for plants (Zandalinas *et al*., 2018).

In particular, in *P. latifolia*, temperature strongly impacted the phenylpropanoid accumulation in leaves, thus suggesting for this species that the investment of assimilated carbon in antioxidant compounds is a main adaptive mechanism to hot Mediterranean summers. In addition, the summer increase in ABA is likely to be temperature dependent in this species. Whereas, in *C. incanus* and *P. lentiscus*, temperature may have principally driven changes in photosynthetic pigments throughout a modulation of VAZ/Chl_Tot_ and Chl*a*/Chl*b* ratio, respectively. Finally, temperature is likely to positively influence the pool of xanthophyll cycle pigments in all species, leading to changes in NPQ and DES which allow flexible and non-flexible thermal dissipation under prolonged environmental stresses. In addition, changes in leaf morphology during the growing season can buffer the effect of the physiological responses to temperature according to species specific leaf habit (Gratani *et al*., 2018).

In conclusion, our results suggest that air temperature may have a greater impact on the performances of Mediterranean coastal dune vegetation when compared with precipitation. Considering the predicted increase in both regional (Fischer and Schär, 2010) and global temperatures (Allen *et al.*, 2015), monitoring heat-responsive traits would allow to identify differences in stress-responses among species. In particular, the study of ecophysiological and biochemical differences among coexisting Mediterranean plants is of utmost importance for the correct understanding of the different selective pressures that this type of vegetation is and is going to be subjected. This could have important implications for understanding plant community dynamics and for the development of conservative strategies of coastal dune plants aimed to preserve their persistence under environmental changes.

## Supplementary Material

Fig_s1_coz070

Figure_S2_coz070
