# Supplementary figures and images for: Seasonal and daily variations in primary and secondary metabolism of three maquis shrubs unveil different adaptive responses to Mediterranean climate
Source: Conserv Physiol. 2019 Nov 5;7(1):coz070. doi: 10.1093/conphys/coz070 (PMC7245392; doi:10.1093/conphys/coz070)

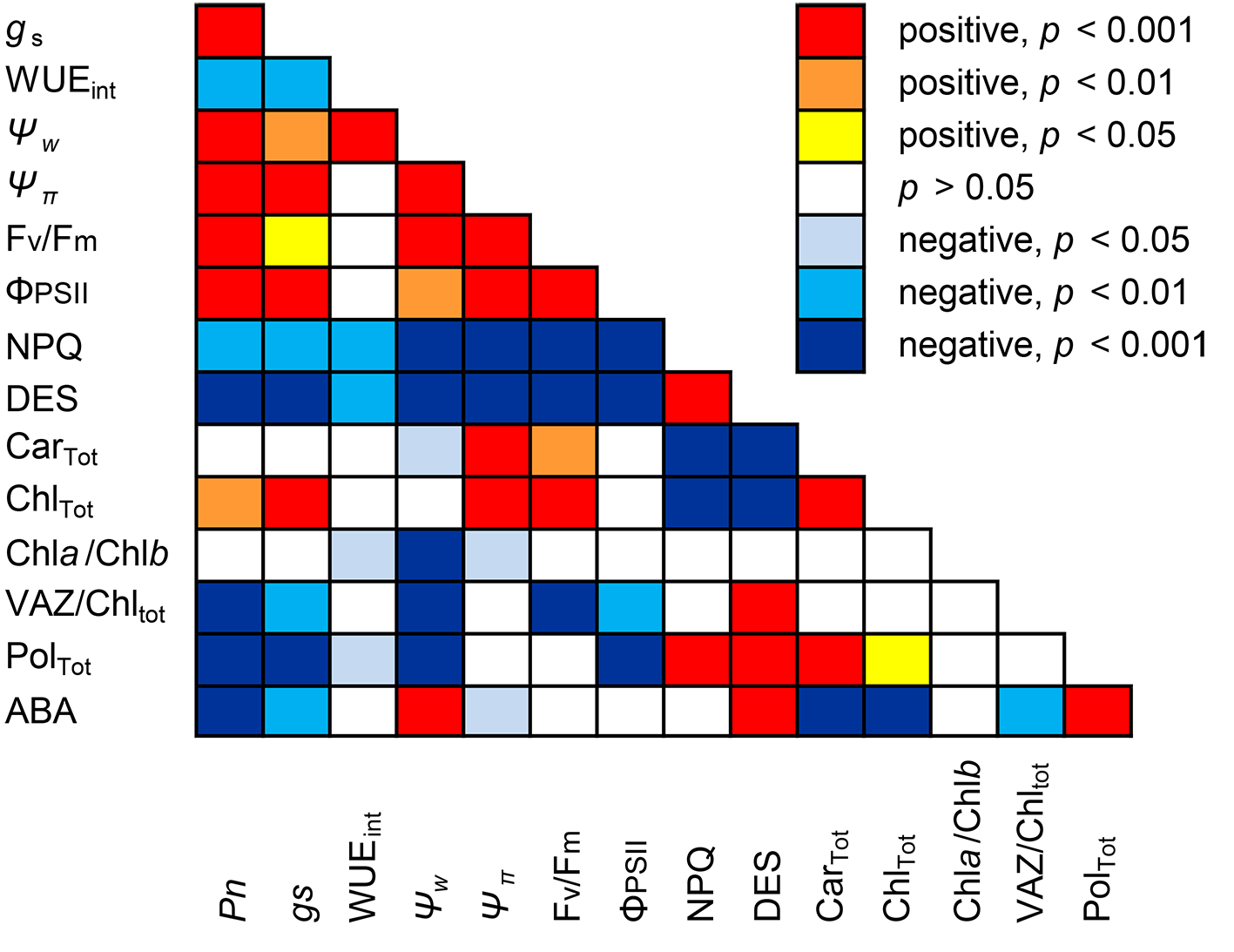

Supplement: Fig_s1_coz070 [file fig_s1_coz070.zip › Fig_s1_coz070.tif]

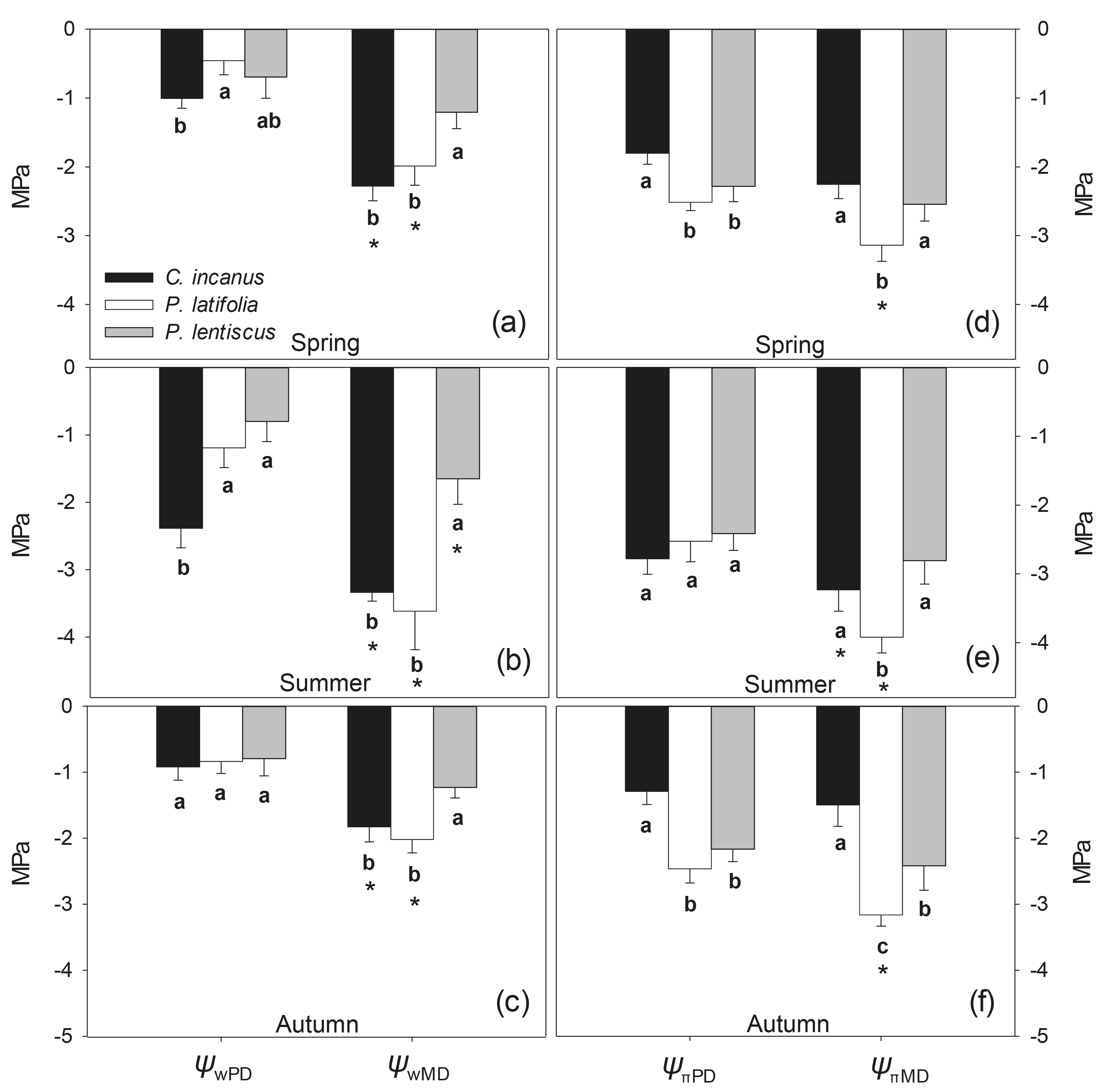

Supplement: Figure_S2_coz070 [file figure_s2_coz070.png]
